# Supplementary material for: Activated NK cells cause placental dysfunction and miscarriages in fetal alloimmune thrombocytopenia
Source: Nat Commun. 2017 Aug 9;8:224. doi: 10.1038/s41467-017-00269-1 (PMC5550461; doi:10.1038/s41467-017-00269-1)
Supplement: Supplementary file 2 — Supplementary Information [file 41467_2017_269_MOESM2_ESM.pdf]

File name: Supplementary Information
Description: Supplementary figures

File name: Peer Review File
Description:

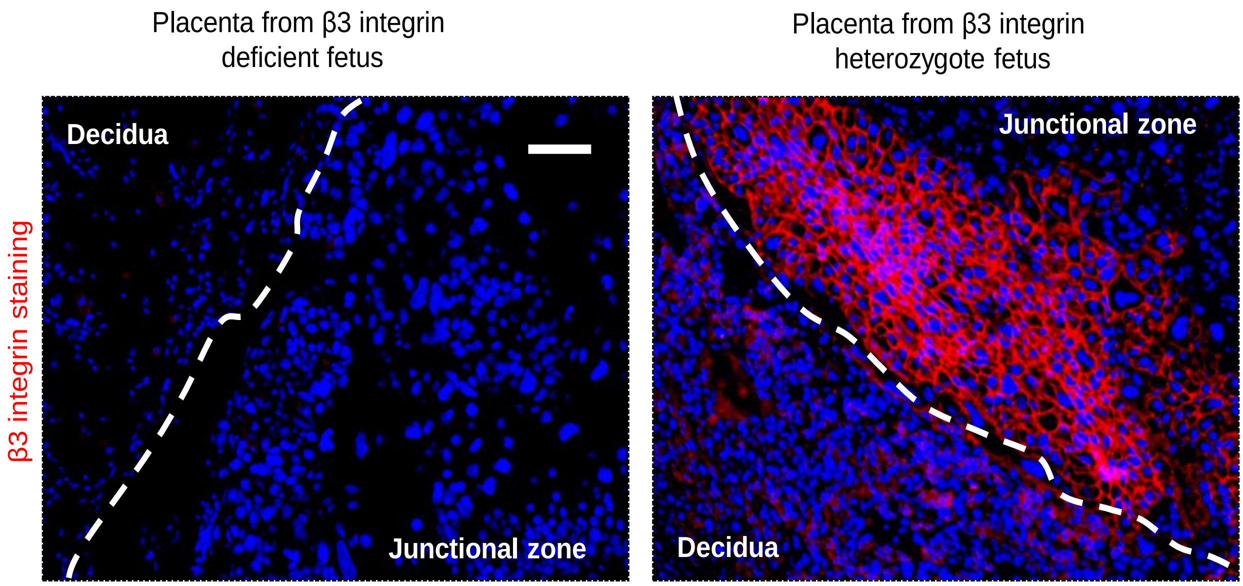

Supplementary figure 1: Phenotyping of the fetuses. The placenta of offsprings from immunized β3$^{-/-}$ female bred with β3$^{-/+}$ mice were phenotyped by immunohistochemistry. Placentas of heterozygote fetuses (β3$^{-/+}$) expressed β3 integrin in the junctional zone whereas placentas of β3$^{-/-}$ fetusses were negative. Scale bars: 50 µm.

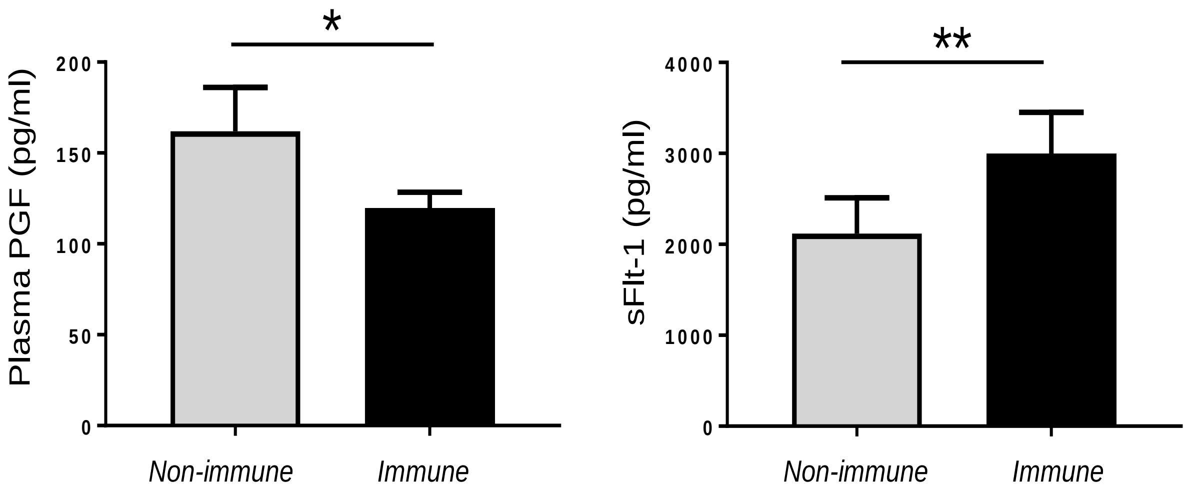

Supplementary figure 2: angiogenic factors in pregnant mice at E14.5. Plasma placental growth factor (PGF) and soluble fms-like tyrosine kinase-1 (sFlt-1) were detected by ELISA. The plasma of *immune* pregnant mice had less PGF, more sFlt-1 than plasma from *non-immune* pregnant mice. Data were collected from more than 6 pregnancies per group. Mean ± SEM. * $p<0.05$ and ** $p<0.01$.

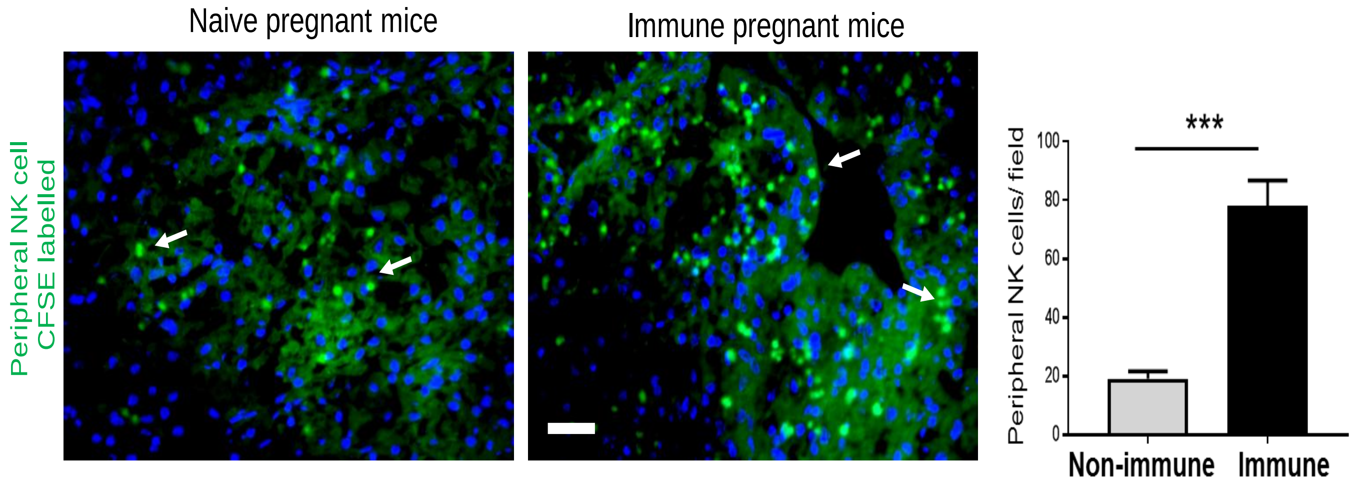

Supplementary figure 3: Peripheral NK cell accumulation in the placenta. Peripheral NK cells were purified by positive selection using anti-NKp46 antibody and cells were labelled with cell tracer fluorescence dye (5-(and-6)-Carboxyfluorescein Diacetate, Succinimidyl Ester, CFSE). Frozen sections of the placentas from mice injected with pNK labeled cells were analyzed. pNK cells significantly accumulated in the placentas of *immune* mice n=3 mice/ group. *** p=0.001. Scale bars: 50 µm.

Flow cytometry gating strategy

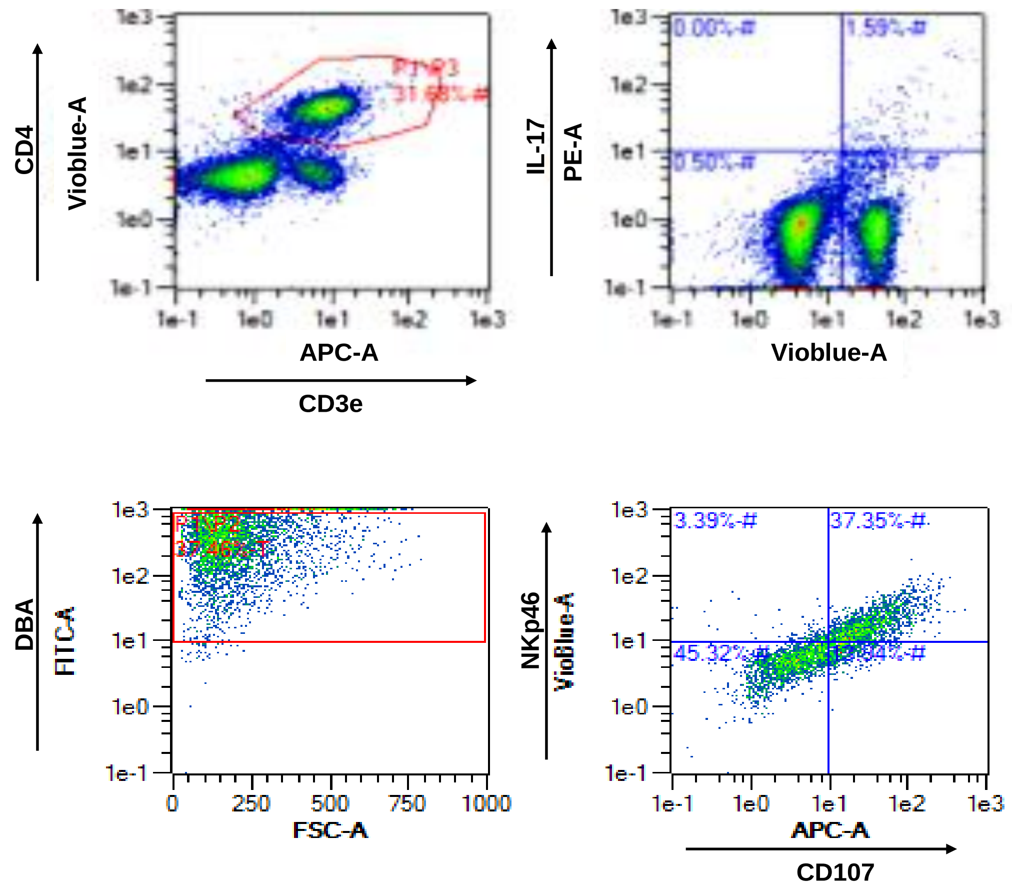

Supplementary figure 4: A) Single cells were gated for CD4 (Vioblue) and CD3e (APC) to select double-positive cells. Then these double–positive cells were gated on PE for IL-17. The percentage of CD4^{+}CD3e^{+} IL17^{+} cells corresponds to triple positive cells from the total splenocyte preparations. **B)** Single cells were gated FITC to select DBA^{+} cells, then these cells were gated on Vioblue and APC to select NKp46 and CD107 double–positive cells

B) Uterine NK cell activation markers (referring to figure 3C)

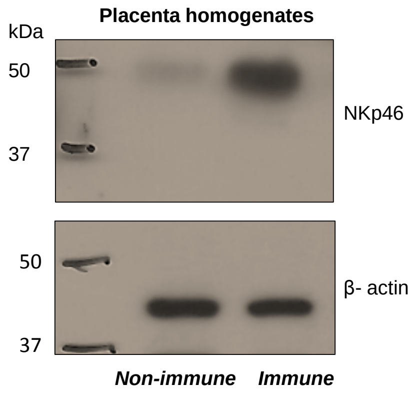

A) Expression of β3 integrin by trophoblast cell lines and mouse placenta (referring to figure 4B)

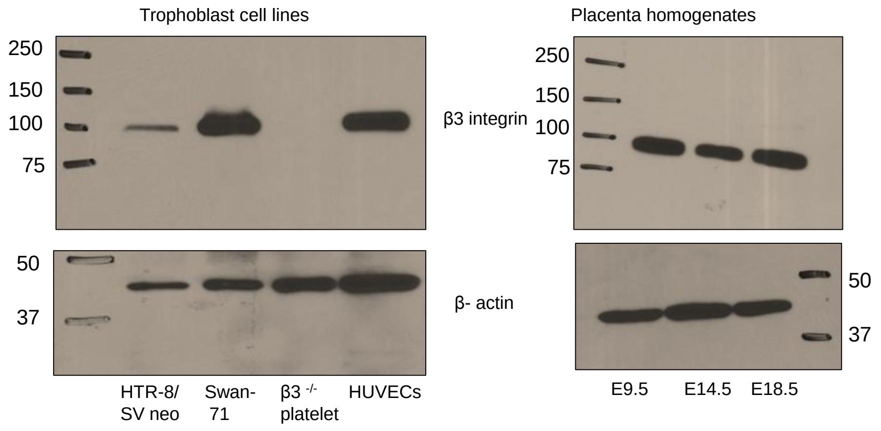

B) NKp46 signaling (referring to figure 5B)

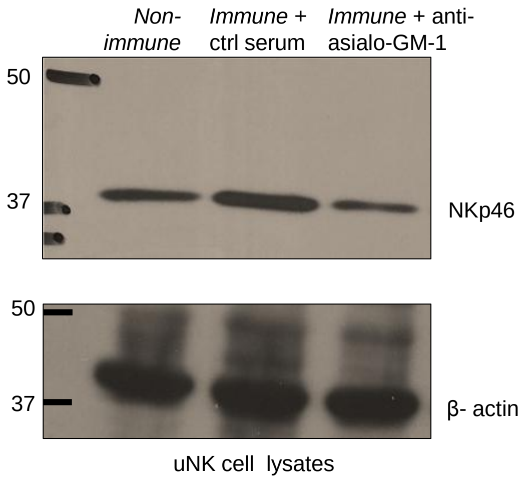

Supplementary figure 5: full-size western blots from figures 3C, 4B and 5B representing uncut blots with marker lines.
